# Involvement of Amylin and Leptin in the Development of Projections from the Area Postrema to the Nucleus of the Solitary Tract

**DOI:** 10.3389/fendo.2017.00324

**Published:** 2017-11-28

**Authors:** Kathrin Abegg, Andreas Hermann, Christina N. Boyle, Sebastien G. Bouret, Thomas A. Lutz, Thomas Riediger

**Affiliations:** ^1^Institute of Veterinary Physiology, University of Zurich, Zurich, Switzerland; ^2^Center for Integrative Human Physiology, University of Zurich, Zurich, Switzerland; ^3^Developmental Neuroscience Program, The Saban Research Institute, University of Southern California, Los Angeles, California; ^4^Inserm U1172, Jean-Pierre Aubert Research Center, University of Lille II, Lille; ^5^Center for Endocrinology, Diabetes and Metabolism, Children’s Hospital Los Angeles, Los Angeles, California

**Keywords:** neuronal development, hindbrain, neuronal programming, energy homeostasis, obesity

## Abstract

The area postrema (AP) and the nucleus of the solitary tract (NTS) are important hindbrain centers involved in the control of energy homeostasis. The AP mediates the anorectic action and the inhibitory effect on gastric emptying induced by the pancreatic hormone amylin. Amylin’s target cells in the AP project to the NTS, an integrative relay center for enteroceptive signals. Perinatal hormonal and metabolic factors influence brain development. A postnatal surge of the adipocyte-derived hormone leptin represents a developmental signal for the maturation of projections between hypothalamic nuclei controlling energy balance. Amylin appears to promote neurogenesis in the AP in adult rats. Here, we examined whether amylin and leptin are required for the development of projections from the AP to the NTS in postnatal and adult mice by conducting neuronal tracing studies with DiI in amylin- (IAPP^−/−^) and leptin-deficient (ob/ob) mice. Compared to wild-type littermates, postnatal (P10) and adult (P60) IAPP^−/−^ mice showed a significantly reduced density of AP-NTS projections. While AP projections were also reduced in postnatal (P14) ob/ob mice, AP-NTS fiber density did not differ between adult ob/ob and wild-type animals. Our findings suggest a crucial function of amylin for the maturation of neuronal brainstem pathways controlling energy balance and gastrointestinal function. The impaired postnatal development of neuronal AP-NTS projections in ob/ob mice appears to be compensated in this experimental model during later brain maturation. It remains to be elucidated whether an amylin- and leptin-dependent modulation in neuronal development translates into altered AP/NTS-mediated functions.

## Introduction

Neuronal programming is a phenomenon that affects the development and maturation of neurons and their projection pathways in the nervous system. Hormonal and nutritional factors involved in energy balance influence the development of hypothalamic brain circuits affecting the maturation of neuronal networks controlling body weight and metabolism during adulthood. One of the best characterized hormones in this context is leptin, which acts *via* the hypothalamic arcuate nucleus (ARC) and other brain sites to inhibit feeding and increase energy expenditure (1, 2). During postnatal life, leptin exerts neurotrophic action required for the development of neuronal projections from the ARC to the hypothalamic paraventricular nucleus (PVN). The outgrowth of these neuronal fibers is triggered by a postnatal leptin surge (3) representing the critical developmental signal. The development of ARC–PVN projections is disrupted in leptin-deficient (ob/ob) or leptin receptor-deficient mice (db/db), but can be rescued in ob/ob mice by postnatal leptin replacement (4, 5). Hypothalamic brain development is also permanently impaired in leptin-resistant diet-induced obese (DIO) rats, and leptin’s ability to promote ARC neurite outgrowth *in vitro* is reduced in the offspring of DIO dams (6).

Another hormone that is thought to exert a neurotrophic action is the pancreatic beta-cell peptide amylin, also termed islet amyloid polypeptide (IAPP), which is coreleased with insulin and acts *via* the area postrema (AP) of the brainstem to inhibit food intake and gastric emptying (7, 8). Amylin directly excites AP neurons (9) which activate downstream projection sites in the nucleus of the solitary tract (NTS) and the lateral parabrachial nucleus (LPBN) (10). The functional amylin receptor results from coexpression of the calcitonin receptor with receptor activity modifying protein (11, 12). Unlike ob/ob mice, amylin-deficient (IAPP^−/−^) mice show a less marked phenotype with respect to food intake, body weight, and glucose homeostasis. While average food consumption does not differ compared to wild-type controls, IAPP^−/−^ mice develop significantly increased body weight from the age of 18 weeks (13). Glucose tolerance and plasma insulin levels are increased in male IAPP^−/−^ following intravenous or oral glucose tolerance tests, which is probably due to deficient autocrine inhibition of insulin secretion from beta-cells (13).

Recent evidence suggests an effect of amylin on brain development and neurogenesis. Postnatal amylin treatment enhances the development of ARC–PVN projection in neonatal rats selectively bred to develop diet-induced obesity (14, 15), and in the AP of adult rats, amylin upregulates the expression of genes involved in neurogenesis and promotes cellular proliferation and differentiation into neurons (15, 16). While the AP/NTS region not only mediates amylin but also leptin-dependent effects on energy homeostasis (17, 18), it is unknown whether endogenous amylin and leptin are required for the development of neuronal projections originating from the AP. To address this, we analyzed the fiber density of AP–NTS projections in postnatal and adult IAPP^−/−^ and leptin-deficient ob/ob mice using the lipophilic tracer DiI?(1,1′-dioctadecyl-3,3,3′,3′-tetramethylindocarbocyanine perchlorate) implanted into the AP. We also measured postnatal amylin levels in wild-type mice to investigate if amylin circulates in postnatal mice at similar levels as in adult mice.

## Materials and Methods

### Animals

Heterozygous (IAPP^+/−^) breeding pairs on a C57BL/6 background were generated from frozen embryos derived from the original mouse strain developed by Gebre-Medhin et al. (13). Founder animals that were used to generate the breeders were provided by Amylin Pharmaceuticals Inc. (San Diego, CA, USA). Homozygous P10 and P60 IAPP^−/−^ and IAPP^+/+^ (controls) littermates were used for the DiI tracing studies. Experiments were approved by the Veterinary Office of the Canton Zurich, Switzerland (ZH202/2006 and ZH42/2014).

To investigate the influence of leptin on the development of AP–NTS projections, we used ob/ob and wild-type (wt) littermates that were bred at the animal facility of the Saban Research Institute (P14 ob/ob mice) or purchased from The Jackson Laboratory (adult P60 ob/ob mice). Experiments were approved by the Institutional Care and Use Committee of Children’s Hospital of Los Angeles (#230-07).

The postnatal time points (P10 and P14) were adapted from previous studies (4) using P10–P16 animals for similar tracing studies. For our studies with ob/ob mice, we chose P14 because the postnatal leptin surge occurring in wild-type animals is completed around this time point (3). Genotypes of ob/ob mice were confirmed by The Jackson Laboratory. Standard PCR was used to genotype amylin-deficient mice. Genotype-specific pairs of primers were used resulting in a 200-bp (IAPP^−/−^) and 107-bp (IAPP^+/+^) amplicon; heterozygous animals showed both bands. The 5′–3′ primer sequences were the following: CTTGGGTGGAGAGGCTATTC and CACAGCTGCGCAAGGAAC for IAPP^−/−^; GTAGCAACCCTCAGATGGAC and GAGGACTGGACCAAGGTTGT for IAPP^+/+^, respectively.

### Perfusion, Brain Preparation, and DiI Implants

Adult mice were deeply anesthetized with an intraperitoneal (IP) injection pentobarbital sodium (200 mg/kg). For mouse pups, tribromoethanol (0.15–0.2 ml/pup, i.e., 4.8–6.4 mg) was used. A small incision with a micro scissor was made into the left cardiac ventricle to facilitate the insertion of a blunt 26 G cannula; after fixing the cannula with a mini-clamp, the right atrium was cut. Exsanguination was then performed by infusing a 0.9% NaCl solution into the left ventricle with a hydrostatic pressure of 14,710 Pa (1,500 mm hydrostatic head) for 2 min, followed by the perfusion with ice-cold paraformaldehyde solution (PFA, 4%) for 6 min. After fixation, brains were immediately transferred into cold PFA and kept for 10 days at 4°C for postfixation.

The cerebellum was carefully dissected with two micro forceps under a stereomicroscope to expose the dorsal surface of the AP. A DiI crystal (Molecular Probes, Eugene, OR, USA), which had been previously selected for size and shape, was then implanted into the AP. Selected crystals had a round, symmetric shape and were 10 and 15 µm in diameter for P10 (IAPP)/P14 (ob/ob) and P60 (IAPP and ob/ob) mice, respectively. The crystal was first placed at the intended implantation site on the surface of the AP, using a micromanipulator. It was then carefully pushed into the tissue with an insect pin. The implantation site was in the center of the dorsal AP surface, and the crystal was placed approximately 50 µm under the ependymal surface. For tracer migration, brains were stored for 10 days in 4% PFA at 37°C in the dark.

Hindbrains were then embedded in 3% agarose. After trimming and fixing the agarose blocks with superglue, a vibratome was used to cut 80-µm thick coronal hindbrain sections corresponding to *bregma* −7.08 and −8.24 mm in the brain of adult mice [according to Paxinos and Franklin’s The mouse brain in stereotaxic coordinates (19)]. Brain slices were kept in KPBS until mounting onto poly-l-lysine-coated glass slides. The slices were covered with 65% KPBS-buffered glycerol before the coverslips were put on the glass slides and fixed with Entellan^®^. Slides were stored at 4°C in the dark to reduce tracer fading.

### Quantification of DiI Fiber Density

Fluorescence microscopy was used to assess successful crystal implantations and symmetric diffusion of the tracer. The following four inclusion criteria had to be met: successful and symmetric crystal implantation into the brain tissue in the midline of the AP and completely under the surface; tracer diffusion area restricted to the AP, and undamaged tissue. To quantify DiI fiber density in the P14 (ob/ob) and P60 (IAPP and ob/ob) mice, one section per brain was scanned with a confocal laser microscope (Leica SP2 AOBS, Leica Microsystems, Wetzlar, Germany). Images (512 × 512 pixels) of the NTS were collected at *bregma* −7.8 or at a corresponding level in postnatal mice to analyze projections from the AP to the NTS. This specific region was chosen because it is caudal to the tracer diffusion field in the AP. Due to the proximity of the NTS and the AP, light emitted by the brightly fluorescent implantation site has a negative influence on the signal to background ratio when images are taken from a level of the NTS that is directly adjacent to the AP. This was avoided by taking images from a more caudal part of the NTS. Image stacks consisted of 75 single images with a thickness of 0.8 µm each.

A wavelength of 543 nm was used for excitation. For the quantification of fiber densities in P10 IAPP^−/−^ and wild-type mice, we used a method that was based on the detection of total fiber length by a software algorithm (see below). While the basic image acquisition was the same for all studies, the confocal signal gain was optimized for this quantification method. Both methods include appropriate controls, but the absolute values of fiber densities in P10 and P60 IAPP^−/−^ and wild-type mice cannot be directly compared.

Image stacks from the P14 (ob/ob) and P60 (IAPP and ob/ob) mice were imported into the imaging software ImageJ, converted to two-dimensional grayscale images, and inverted. After auto-correction of brightness and contrast by the ImageJ auto-correction algorithm, a manual thresholding was done detecting DiI-labeled fibers. The total pixel number of detected fibers was calculated using the software’s histogram function. The person evaluating the images was blinded with respect to details about the experimental group.

Image stacks obtained from P10 IAPP^−/−^ mice and controls were imported into the analysis software Imaris (Version 6.4.0, Bitplane, Zurich, Switzerland). After three-dimensional reconstruction and volume rendering, the filament tracer function was used to analyze fiber density. Briefly, an autopath algorithm was used to generate filaments by connecting starting and end points based on local intensity contrast. The largest diameter of starting points was set to 8 µm, the thinnest of end points to 0.931 µm, spine detection was disabled, and the signal range was adjusted manually by thresholding. Fibers were then skeletonized to a diameter of 1 pixel to allow the software to calculate different parameters, among them the total fiber length in the entire volume of the image stack.

### Measurement of Postnatal Amylin Levels

Blood samples from wild-type mice were taken between P6 and P9 after birth. Mouse pups were anesthetized with an IP injection of tribromoethanol (0.15–0.2 ml for P6–P9 wild-type pups, i.e., 4.8–6.4 mg). The abdomen and the thorax were opened and the heart was carefully exposed. The pericardium was removed and the right ventricle was penetrated with a cannula. Blood was immediately transferred into EDTA tubes. The protease inhibitor aprotinin was added instantly. Blood was cooled on ice until centrifugation. Samples were centrifuged immediately for 10 min at 2,800 rpm to separate blood plasma. Plasma was subsequently transferred into Eppendorf tubes and stored at −20°C until further processing. For the analysis of blood amylin levels, the Mouse Endocrine LINCOplex™ assay was used according to the manufacturer’s instructions (Linco Research, Inc.).

### Statistical Evaluation

All measures of DiI fiber density were expressed as mean ± SEM and statistically compared by Student’s *t*-test. Postnatal amylin values were compared by one-way ANOVA followed by Tukey’s multiple comparisons test.

## Results

**Figure 1:**
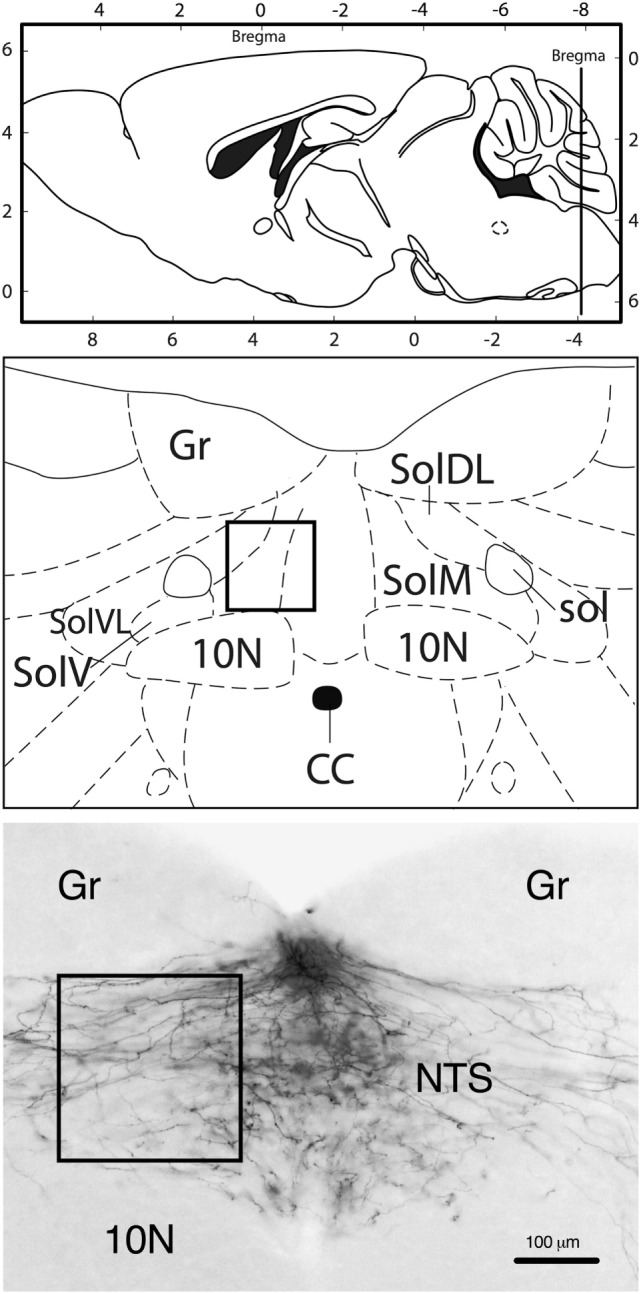
Neuroanatomical localization of the region that was used for quantification (rectangle). Approximate rostrocaudal level of the *nucleus of the solitary tract* (NTS): bregma −7.8 mm (caudal of the DiI crystal implantation site in the area postrema). Representative image (10-fold magnification) of DiI staining illustrating that labeling was only present in fibers, but not in neuronal cell bodies. 10N, vagus nerve nucleus; CC, central canal; Gr, gracile nucleus; sol, solitary tract; SolDL, solitary nucleus, dorsomedial part; SolM, solitary nucleus, medial part; SolV, solitary nucleus, ventral part; SolVL, solitary nucleus, ventrolateral part. Schematic drawings: modified and reproduced with permission from Ref. (19).

In all animals included in the analysis, the DiI tracer diffusion field was confined to the AP without significant spreading into the adjacent NTS; hence, the NTS only contained DiI-labeled fibers, but no DiI-positive cell bodies (Figure 1). The fibers extended into the NTS, forming a dense plexus covering large parts of the cross-sectional area of the NTS. Most fibers appeared to transverse in diagonal dorsoventral direction from the AP to the NTS.

**Figure 2:**
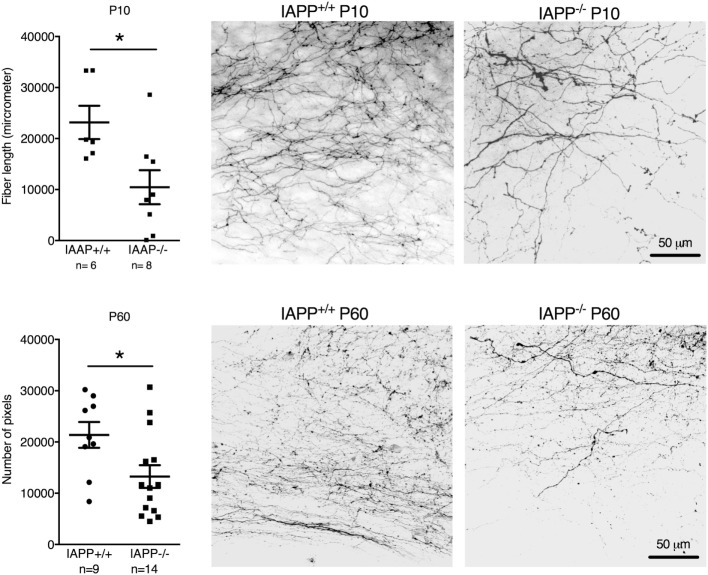
Quantification and representative images of DiI staining of area postrema–NTS projections in P10 (upper panel) and P60 (lower panel) IAPP^+/+^ wild-type and amylin-deficient IAPP^−/−^ mice. Amylin deficiency resulted in reduced fiber densities at both time points compared to controls (*p < 0.05, Student’s *t*-test).

IAPP^−/−^ mice showed a significantly reduced density of AP–NTS DiI-labeled fibers at P10 (*t* = 2.651, df = 12, *p* = 0.0211) and P60 (*t* = 2.372, df = 21, *p* = 0.0273) compared to their respective IAPP^+/+^ littermates (Figure 2). Even though the absolute values of AP–NTS fiber density cannot be directly compared, the relative difference between the genotypes was profound and significant at each time point (P10: 55% reduction; P60: 38% reduction). Hence, amylin deficiency leads to a deficit in AP–NTS projections in postnatal mice that persists until adulthood.

**Figure 3:**
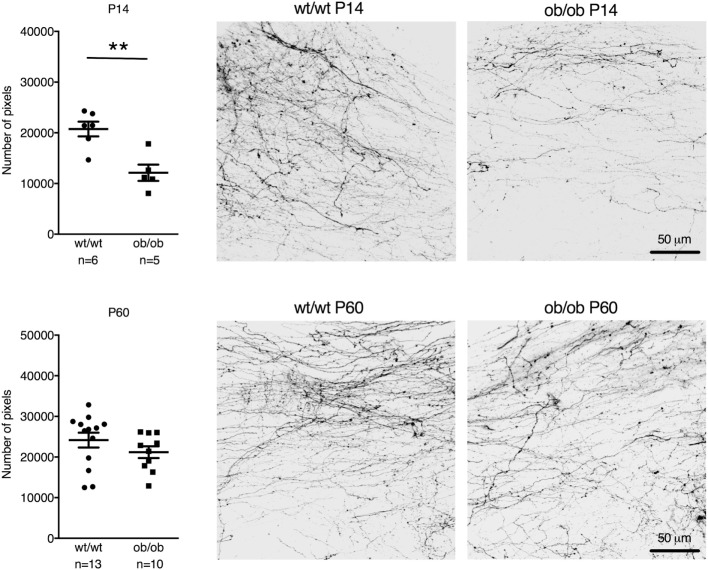
Quantification and representative images of DiI staining of area postrema–NTS projections in P14 (upper panel) and P60 (lower panel) wt/wt and leptin-deficient ob/ob mice. Leptin deficiency resulted in reduced fiber densities compared to controls in P14 but not P60 mice (**p < 0.01, Student’s *t*-test).

Leptin-deficient ob/ob mice also showed reduced postnatal AP–NTS DiI-labeled fiber density at P14 (reduction by 42%; *t* = 3.981, df = 9, *p* = 0.0032). However, in contrast to IAPP^−/−^ mice, adult ob/ob mice had a similar fiber density as wild-type controls (Figure 3).

**Figure 4:**
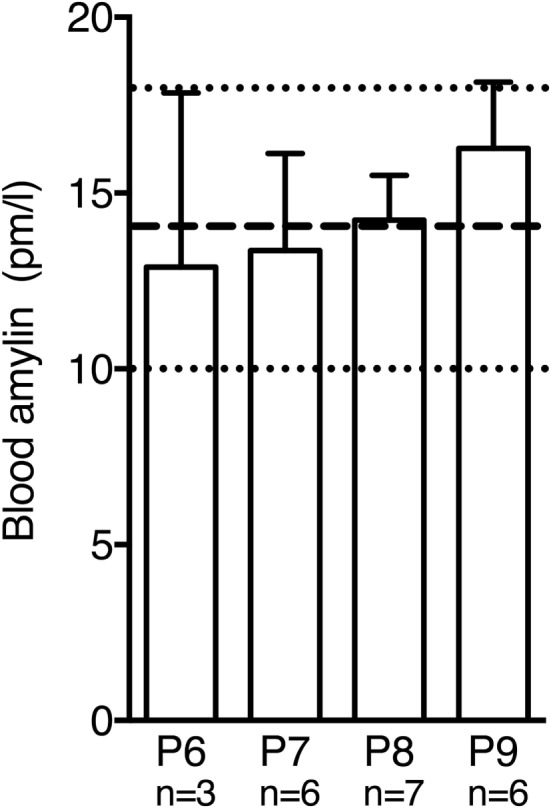
Postnatal amylin plasma levels in wild-type mice did not differ significantly between P6 and P9. During this time window amylin levels were similar to values reported for adult mice [dashed line and dotted lines represent mean ± SEM reported in Ref. (20)].

Circulating plasma amylin levels did not differ significantly between P6 and P9 in wild-type mice [Figure 4; *F*(3,18) = 0.3765, *p* = 0.7710]. Moreover, values were comparable to amylin levels measured in adult mice without amylin deficiency (20).

## Discussion

Our findings extend the concept that amylin and leptin exert a neurotrophic action required for the perinatal maturation of brain circuits involved in energy homeostasis. In contrast to leptin’s effect on hypothalamic development, little is known about the maturation of neuronal AP–NTS projections and the possible importance of hormonal signals in this process. We now demonstrate a crucial role of amylin for the development of AP–NTS projections that are likely to control ingestive behavior and gastrointestinal function during adulthood.

The ontogenetic development of mammals is characterized by fundamental challenges regarding nutritional supply and behavioral control mechanisms. The amount of milk ingestion in rats until postnatal day 10 primarily depends on olfactory and tactile cues promoting nipple attachment and the availability of milk, but not internal cues reflecting the nutritional state (21). Pre- and post-absorptive cues that control ingestive behavior start to develop after P10 to influence suckling behavior and total milk intake (22–24), however, newborn rats are capable of independent feeding by licking milk (25, 26). In contrast to suckling-related milk intake, independent feeding can be inhibited in neonatal rats by exogenous CCK (25, 26). Hence, the neuronal substrates mediating satiation *via* the brainstem are functional at birth. While the CCK effect is unrelated to the neurotrophic actions observed in our current study, these behavioral findings indicate a prenatal development of the neuronal circuitry of the dorsal vagal complex. This prenatal development is further supported by neuroanatomical studies (27) and contrasts with the postnatal maturation of hypothalamic circuits (28).

We identified amylin as a crucial hormonal factor for the maturation of AP–NTS projections. The neuronal projections between the AP and the NTS are highly developed already at P10. We did not try to identify the time point that marks the beginning of amylin’s neurotrophic action. Based on the considerations above, we consider it likely that amylin acts prenatally to promote the outgrowth of AP–NTS projections, but we cannot exclude neurotrophic effects occurring after birth. While pancreatic amylin expression has been demonstrated in mice at embryonic day 12 and in humans at gestational week 18 (29), prenatal plasma levels of amylin are unknown. To the best of our knowledge, there are no published data on plasma amylin levels of rodents at time points earlier than our measurements between P6 and P9. Between these time points, amylin levels did not change and were similar to the plasma concentration of adult mice (20). We cannot exclude that a temporary prenatal or an early postnatal (P0–P5) amylin surge functions as a developmental signal, similar to the postnatal leptin surge (3). However, we believe that an amylin surge is not a necessary pre-requisite for an amylin-dependent neurotrophic function. In fact, we consider this possibility unlikely based on the glucose-dependent cosecretion of amylin with insulin. A surge of pancreatic amylin (and insulin) secretion that is strong and long enough to induce neuronal maturation could impair glucose homeostasis after birth. It appears more plausible that physiological changes in amylin levels or possibly an increase in postnatal basal amylin levels as a consequence of suckling play a more relevant role. Amylin is also expressed in the rat and human placenta (30), but it is unknown whether placental amylin might reach the embryo in effective amounts. Furthermore, it is unknown whether amylin locally expressed in the brain could be implicated in the effects observed in our study.

Importantly, the disruption of AP–NTS fiber development in IAPP^−/−^ mice persisted until adulthood, which raises the question about possible consequences for the development of obesity and metabolic disease. While IAPP^−/−^ animals are only moderately and transiently overweight compared to wild-type mice, it is difficult to dissociate specific effects related to underdeveloped AP–NTS projections from other amylin-dependent mechanisms that might be defective in these mice (13). Therefore, more specific knockout models (e.g., site-specific amylin receptor deficiency) should be used to investigate the physiological or pathophysiological relevance of developmental deficits in neuronal AP–NTS signaling. It also needs to be elucidated whether the neurotrophic action of amylin in the brainstem is restricted to functional systems controlling energy homeostasis or whether other AP/NTS-related functions, such as cardiovascular modulation (31), could also be affected. In this context, it should also be noted that the technique used in our studies is unable to distinguish AP projections terminating in the NTS and fibers passing through the NTS to other projection sites (e.g., the LPBN).

Similar to IAPP^−/−^ mice, leptin-deficient ob/ob mice also showed reduced postnatal AP–NTS fiber density, but this effect was no longer apparent in adult animals. While our findings do not point to a direct interaction between the neurotrophic effects of amylin and leptin, further studies are required to specifically investigate this possibility. It seems that by P60, the lower density of AP projections in P14 ob/ob mice was compensated for, resulting in a similar fiber density compared to P60 and P14 wt/wt animals. This phenomenon is clearly different from the leptin-dependent action on ARC to PVN projections, which persists until adulthood (4). We can only speculate about the potential underlying reasons. Leptin deficiency leads to obesity and concomitant hormonal changes that might exert a neurotrophic effect on brainstem circuits, e.g., increased levels of amylin and insulin (20, 32), that may perhaps compensate over time for the lack of leptin’s neurotrophic action in ob/ob while the animals gain weight. Similarly, increased signaling of other feeding-related stimuli converging in the AP/NTS region might shape the development of AP–NTS projections until adulthood in ob/ob mice. Finally, it is also possible that apart from the effects on AP–NTS projection density, the functional properties of these neuronal connections might be altered, which is not necessarily reflected by differences in fiber density *per se*. Other parameters, such as the density of synaptic connections between AP efferent and NTS neurons, could be important determinants for adequate AP–NTS neurotransmission. Whether such functional properties are compromised in addition to the neuroanatomical alterations observed in our study remains to be elucidated.

In conclusion, our study revealed neurotrophic actions of amylin and leptin on the connectivity between brainstem feeding centers. These findings substantiate the concept that the developing brain is sensitive and possibly vulnerable to embryonic or perinatal hormonal influences related to energy homeostasis. Possible consequences of this neuronal programming for the risk to develop obesity and metabolic disorders need to be dissociated from the global effects of hormone deficiency in these models.

## Ethics Statement

Experiments were approved by the Veterinary Office of the Canton Zurich, Switzerland, and by the Institutional Care and Use Committee of Children’s Hospital of Los Angeles.

## Author Contributions

Design: KA, AH, CB, SB, TL, TR; experiments: KA, AH, CB, SB, TR; data evaluation: KA, AH, TR; preparation of manuscript: KA, AH, TR; proof-reading: CB, SB, TL.

## Conflict of Interest Statement

The authors declare that the research was conducted in the absence of any commercial or financial relationships that could be construed as a potential conflict of interest.
